# Effects of Bucket Type and Angle on Downstream Nappe Wind Caused by a Turbulent Jet

**DOI:** 10.3390/ijerph16081360

**Published:** 2019-04-16

**Authors:** Jijian Lian, Junling He, Wenjuan Gou, Danjie Ran

**Affiliations:** State Key Laboratory of Hydraulic Engineering Simulation and Safety, Tianjin University, Tianjin 300350, China; jjlian@tju.edu.cn (J.L.); 1017205048@tju.edu.cn (J.H.); 1018205051@tju.edu.cn (D.R.)

**Keywords:** flip bucket, nappe wind, splash, two-phase flow, drag force

## Abstract

The downstream nappe wind caused by flood discharge has a great influence on the rainfall distribution, the operational safety of dams, and their surrounding ecological environments. A physical experiment was conducted to measure the spatial distribution of the downstream nappe wind and the splash for a continuous bucket (CB) and a tongue-shaped bucket (TB) for five bucket angles (40°, 45°, 50°, 55°, and 60°). The experimental results demonstrate that the trajectory width and height of the nappe increase as the angles increase, but the effect on the length is converse. The wind velocity and splash weight of the two buckets decrease along the flowing direction. In the lateral direction, the wind velocity and splash weight for the CB decrease as *y* increases, but the wind velocity of the TB trends to humplike; its splash weight decreases near the axis of the bucket, and is stable in the other region. In the vertical direction, the velocity for the CB increases and then decreases as *z* increases, but that for the TB decreases monotonously. The velocity of the wind and weight of the splash for the CB decreases with the increasing angles, but those of the TB peak at 45°. The findings are useful for the more accurate prediction of rainfall.

## 1. Introduction

**Figure 1:**
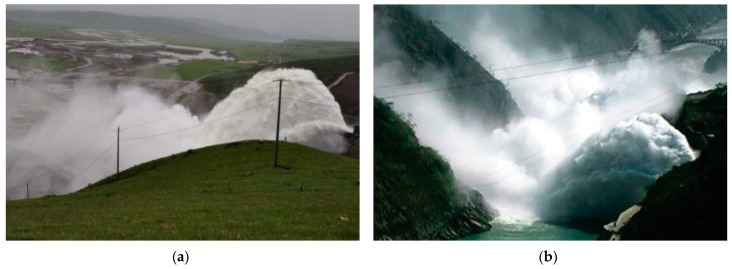
Flood discharge atomization caused by trajectory energy dissipation. (**a**) Nazixia Hydropower Station and (**b**) Dongjiang Hydropower Station.

Hydropower, as a renewable and clean form of energy, takes advantage of its mature technology and stable supply, and plays an important role in the global energy supply. The discharge structure is a great structure for the hydropower station [1,2]. The trajectory energy dissipation that is commonly used in high dams with large flood discharges utilizes the great kinetic energy of the high-speed water flow to discharge the trajectory to the downstream plunge pool, and much energy is consumed by the mixing and friction between the nappe and air [3,4,5]. The trajectory energy dissipation has many advantages. However, when it consumes the energy of the trajectory nappe, it causes the downstream nappe wind and the flood discharge atomization, as shown in Figure 1. The rainfall intensity is far greater than that of the natural rainstorm [6,7]. Su et al. [8] and Liu et al. [9] considered that the downstream rainfall intensity exceeded 500 mm/h in Ertan and Dongjiang hydropower stations, and the intensity would threaten the dam safety and influence the surrounding ecological environment. The downstream nappe wind provides the power for the diffusion of fog and rain and magnifies the coverage of the flood discharge atomization [10,11]. Lian et al. [12] studied the influence of the nappe wind on the coverage of the flood discharge atomization and believed that the length of the storm region increased by 14% when considering the nappe wind in the Hongkou Hydropower Station. Therefore, the spatial distribution of the downstream nappe wind is a crucial issue, and should be clarified urgently in order to ensure the safety of both the dams and the ecological environment around them.

The flip bucket of the discharge structure determines the trajectory nappe. According to the outlet shape, the flip bucket is generally classified as a constant width bucket [13,14] and an expansion bucket [15]. The hydraulic characteristics of trajectory nappe have been extensively studied, such as the height, length [16], energy dissipation [17], and aeration [18] of the trajectory nappe, and the downstream scour [19]. The continuous bucket is the basic type of flip bucket and is the most traditional bucket [20]. Its section is rectangular and remains constant, and the width of the trajectory nappe remains identical. The constant width bucket has a simple structure and cavitation erosion resistance [15], but its scour is relatively serious for the downstream riverbed. The contracted slit bucket deforms the approach flow by a transverse contraction and longitudinal extension at the lip of the flip bucket, increasing the longitudinal diffusion of the trajectory nappe [21,22]. The expansion flip bucket is proposed and its possessive energy of the unit width discharge is reduced by expanding the cross-section of the trajectory nappe. The tongue-shaped bucket aggravates the transverse diffusion of the trajectory nappe under the action of the arc curve at the lip of the bucket [15]. The scour of the downstream riverbed is reduced, and the bottom slab damage of the plunge pool is avoided. The skew bucket changes the impact position of the trajectory nappe by a curved surface attached to a sidewall [5].

The nappe wind is mainly composed of the dragging nappe wind, the diffusing nappe wind, and the splashing nappe wind [23]. When the flood discharges, the high-speed trajectory nappe carries huge kinetic energy, and vortices are generated at the interface of the nappe and air due to turbulence. It causes the momentum exchange between the water and air, and the dragging nappe wind appears. When the trajectory nappe jumps into the air, the high velocity of the nappe causes the diffusion and aeration of the nappe. The turbulence of the nappe causes its breakup, and the breakup nappe exchanges momentum with the air, and the diffusing nappe wind appears. When the nappe impinges into the downstream, a large number of droplets splash around due to the elasticity of the water, which produces an instantaneous impulse action on the surrounding air and forms the splashing nappe wind. Hu et al. [24] established the mathematical model of the wind-driven flow and studied the characteristics of the flow field. Zhang et al. [25] studied the wind waves and atmospheric turbulence using a wind–wave momentum transfer experiment, and found that the profile of the wind speed above waves is logarithmic, and the momentum flux near waves is constant. Zhou et al. [26] selected different drag coefficients in order to simulate the wind-driven flow in an open channel on Taihu Lake, and found that the drag coefficient varied with the wind speed. Li et al. [27] simulated the arrival and evolution of the nappe wind during the flood discharge of a hydropower station, and the results showed that were was a counterclockwise vortex of high-speed wind downward from the dam due to the instantaneous and strong momentum of the high-speed liquid flow, and the small part of the nappe wind was formed by the dragging action at the interface of the water and air, and the nappe wind is mainly caused by the instantaneous impulse action due to splash water droplets. Sha et al. [28] simulated the nappe wind during a flood discharge; they found that the largest nappe wind appeared in the impinging position, and after a certain distance from the impinging position, the wind speed decayed rapidly, and several vortex structures formed behind the dam. Zhang et al. [23] obtained the computational formula of nappe wind based on the instantaneous impulse action. The nappe wind is an extremely complex issue due to its paroxysmal and instantaneous characters and the deformation, breakup, and collision of splashing droplets. A huge challenge exists regarding the theory and numerical simulation. A prototype observation is also difficult to obtain because of the harsh environment in heavy rainfall areas. The physical experiment should be conducted as a main way to study this issue.

The interaction of water–air two-phase flows has been studied a lot, and researchers always focus on the characteristics of the water, but the analysis of the nappe wind that is generated by the trajectory nappe has received relatively less attention. A physical experiment was conducted in order to study the detailed spatial distribution of the downstream nappe wind using a continuous bucket (CB) and a tongue-shaped bucket (TB) with five different bucket angles (40°, 45°, 50°, 55°, and 60°). This research finding can help provide the empirical value for the stochastic model of flood discharge atomization in order to accurately simulate the rainfall intensity distribution and improve the safe operations of high dams.

## 2. Experimental Setup

### 2.1. Experimental Method

**Figure 2:**
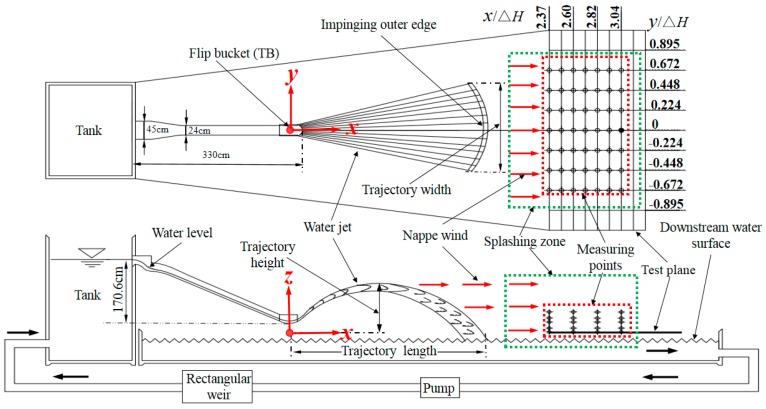
Schematic diagram of the experiments and measuring point arrangement as an example of a tongue-shaped bucket (TB).

Figure 2 depicts the schematic diagram of the experiments and the measuring point arrangement as an example of TB. In order to more conveniently describe the distribution of nappe wind, the point of the lowest level along the axis of the spillway was projected onto a measurement plane as an origin, in which the flowing direction is the *x*-axis, the lateral direction is the *y*-axis, and the vertical direction is the *z*-axis, as shown in Figure 2.

The physical model system mainly consists of a spillway with a flip bucket and an upstream water tank that is connected to a downstream pool by a pipe with a pump and a rectangular weir. A recirculating flume is adopted to supply water for the experiment system. The spillway was fixed at the upstream tank 1.88 m above the test plate. The upstream water tank has a length of 10 m, a width of 10 m, and a height of 6 m. The upstream water tank is equipped with a stabilizer in order to ensure a smooth flow. The downstream pool has a length of 9.5 m, a width of 4.2 m, and a height of 0.2 m. The vertical level of the flip bucket is 32.8 cm, and the downstream water level *z* is –20 cm. The discharge structures are made of Plexiglass. The outlet velocity is 4.26 m/s as measured by a Pitot tube, and the outlet flow rate is 56.6 L/s, as measured by a rectangular weir. The water level difference (Δ*H*) between upstream and downstream is 223.4 cm, which is controlled by a pipe valve and a downstream tailgate, respectively, and the water level error is ±1 mm. The flow rate, outlet velocity, and hydraulic parameters of the trajectory nappe are necessarily measured at this stable water level.

The measuring points of the nappe wind and splashing weight are arranged at the grid intersection of the test plane, and the test plane is regarded as the datum elevation, as shown in Figure 2. The water-level difference has a great influence on the downstream nappe wind and splashing weight distribution for the transformation of the spatial distances to a non-dimensional parameter. The downstream nappe wind is measured using a QYCG-09 ultrasonic wind sensor (Produced by Qingyi Electronic Technology Co., Ltd.), and its maximal measuring range is approximately 0 to 10 m/s with a measuring accuracy of 0.2 m/s ± 0.02 × *v* (*v* is the actual wind velocity). The QYCG-09 ultrasonic wind sensor is composed of four ultrasonic transducers. The functions of the transducers include acoustic transmission and reception. The distance between the transducers is 200 mm. The ultrasound wind speed sensor utilizes the ultrasonic time-difference method to measure the wind speed. In this research, each point was measured with the exposure time of 400 s and an acquisition frequency of 1 Hz. The experiment was carried out at 25 ± 1 °C, and the relative humidity was 30 ± 1%. Experiments were conducted and 400 data points were collected with the interval of 1 s for each measuring point and each condition, and the average value was regarded as the magnitude of the nappe wind velocity. The splashing weight was measured by using sponge collection boxes with a length of 20 cm and a width of 10 cm, which were numbered and arranged at the downstream test plane. The boxes were weighed using an electronic scale with the accuracy of 0.001 g. The numbered sponge boxes were arranged point by point from the downstream far boundary of the splashing area. They were collected 80 min later, and then the weight of the splashing droplets was measured. The result was the average of three test data. The maximum length and width of the trajectory nappe were measured by a ruler, and the maximum height of the trajectory nappe was measured by a level gauge. The maximum value over 3 minutes was observed and recorded, and the average of the three values was the result.

### 2.2. Test Conditions

The type of the flip buckets for trajectory energy dissipation is divided into the constant width type and the expansion type, and the continuous bucket and the tongue-shaped bucket are the typical shape of the constant width type and the expansion type, respectively. The two buckets have features of simple construction and good cavitation resistance, and they are widely used in hydropower stations. Therefore, studying the spatial nappe wind distribution and the rainfall intensity distribution of the continuous bucket and the tongue-shaped bucket is urgent in hydraulic engineering.

**Figure 3:**
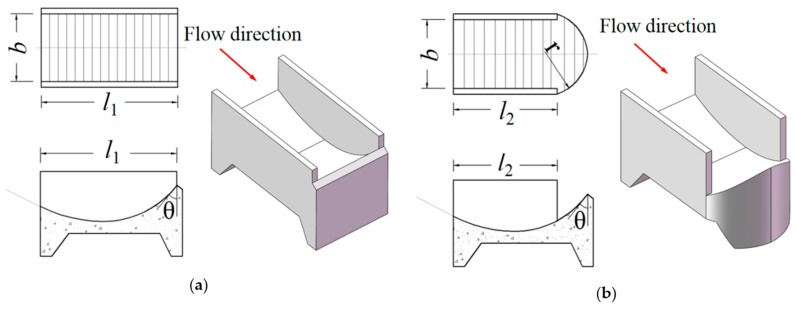
Flip-bucket types for the testing: (**a**) continuous bucket (CB) and (**b**) tongue-shaped bucket (TB) (*θ* represents the bucket angles, *l*_1_ represents the length of the flip bucket, *b* represents the width of the flip bucket, *l*_2_ represents the sidewall length of the TB, and *r* represents the radius of the TB).

The experiment was conducted in order to study the spatial distribution of the downstream nappe wind with the two types of buckets under five different bucket angles. The shapes of the CB and TB are shown in Figure 3, where *θ* represents the bucket angle (40°, 45°, 50°, 55°, and 60°); *b* represents the width of the flip bucket, and its value is 24 cm; *l*_1_ represents the length of the CB, and its value is 48.38 cm; *l*_2_ represents the sidewall length of the TB, and its value is 37.41 cm; and *r* represents the radius of the TB, and its value is 15.48 cm.

## 3. Results and Analysis

### 3.1. Hydraulic Characteristics of the Trajectory Nappe

**Figure 4:**
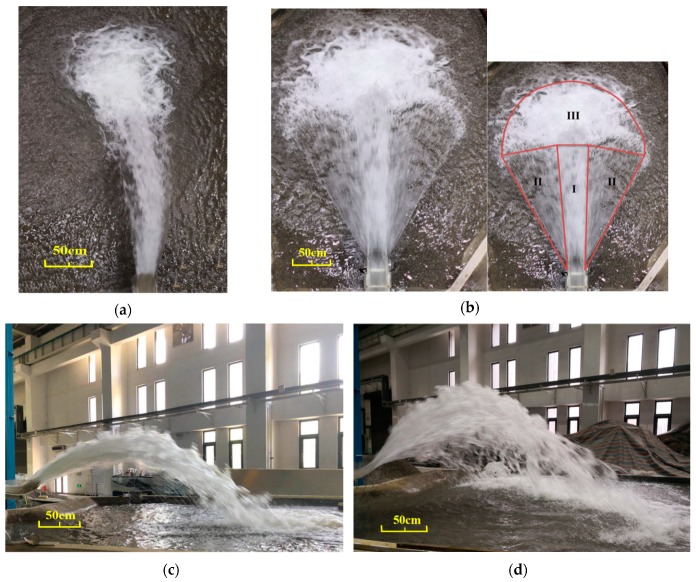
Photographs of the flow regime of the trajectory nappe under the same flow conditions (bucket angle: 50°): (**a**) the top view of the CB, (**b**) the top view of the TB, (**c**) the side view of the CB, and (**d**) the side view of the TB.

The flip bucket’s characteristics have a great influence on the hydraulic characteristics of the trajectory nappe. The flow patterns of the CB and TB with a bucket angle of 50° were obtained at a flow discharge of 56.60 L/s with an outlet velocity of 4.26 m/s, as shown in Figure 4. The nappe flows through the flip bucket, jumps into the air, and then directly impinges on the water surface of the downstream. Due to the shape of the bucket, the trajectory nappe of the CB shape is similar to a crescent, and is more concentrated than that of the TB, as shown in Figure 4a,c. The width of the CB’s nappe is similar to the width of the bucket, but the TB’s nappe is fan-shaped, and the width of the nappe increase with the movement of the nappe, as shown in Figure 4b,d. The nappe of the TB is composed of three regions. Region I is the core of the nappe; the width of this region is almost the same as the width of the bucket, and the outflow angle of the nappe remains the same as the bucket angle. Region II is the diffusion of the nappe; in this region, the outflow angle is smaller than that of the bucket due to the shorter length of the wall and the circular edge of the bucket, and the diffused nappe having less trajectory height, but a greater trajectory length. Region III is the impinging area; the impinging area of the CB is larger than that of the TB. The main reason for the different shapes is that the outlet regime of the TB is circular, and the lengths of the sidewalls of the two buckets are different. The length of the sidewall of the CB is 48.38 cm and that of the TB’s length is 37.41 cm, although the lengths of the two buckets are both 48.38 cm. Therefore, a circular outlet with shorter sidewalls benefits the diffusion of the nappe.

**Figure 5:**
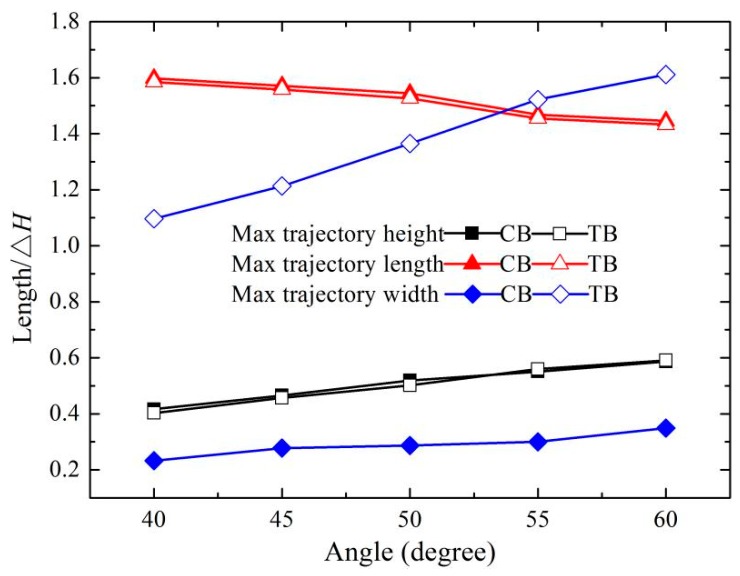
Hydraulic characteristics of the CB and TB at different bucket angles.

The hydraulic parameters of the trajectory nappes at the CB and TB with the various bucket angles under the constant discharge condition are shown in Figure 5, where Δ*H* is the difference between the upper and lower water level. The trajectory nappe heights of the CB and TB are similar, and range from 0.40 to 0.59. The trajectory nappe lengths of the CB and TB decrease with the increase of the bucket angle, and the trajectory nappe length of the CB is slightly larger than that of the TB. The width of the impinging outer edge of the TB is roughly four times that of the CB, and the maximum difference is as high as 1.26. The width and the height of the trajectory nappe at both buckets gradually raise with the increase of the bucket angles, but the nappe length decreases. These results are in good agreement with the previous research [16,29]. Compared with the CB, the trajectory nappe of the TB is more diffusive, and the air resistance and the dynamic energy dissipation of the nappe are relatively enhanced, and the trajectory nappe length relatively decreases.

### 3.2. Effects of Bucket Shapes on Wind Velocity

**Figure 6:**
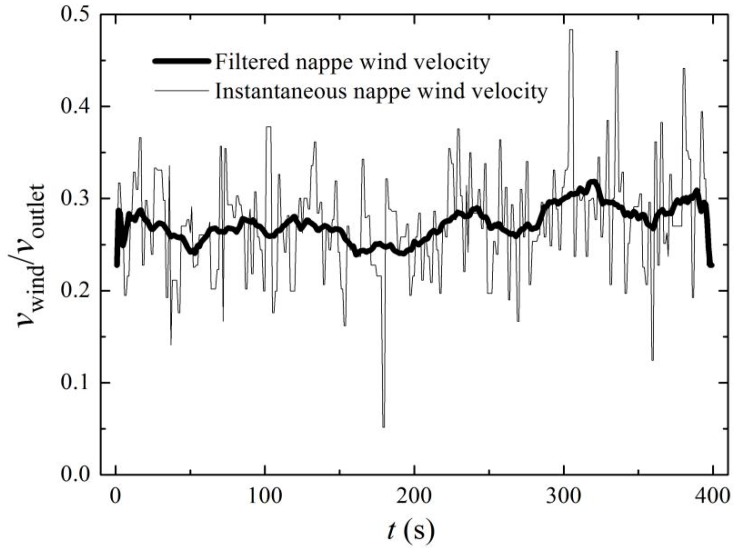
Time history curve of the nappe wind of the CB at 50° and (3.04, 0, 0).

The spatial distribution of the downstream nappe wind has a great influence on the intensity and range of the rainfall, and is closely related to bucket shape. In this paper, it is necessary that the relationship between the spatial nappe wind distributions and the shapes of the buckets at five bucket angles are clarified. Figure 6 depicts the time history curve of the nappe wind of the CB at the measurement point (3.04, 0, 0), and it is smoothed using the moving average method [30]. A total of 400 experimental data points were collected with the interval of 1 s for each measuring point, and the average value is regarded as the magnitude of the nappe wind velocity.

#### 3.2.1. Horizontal Distribution

**Figure 7:**
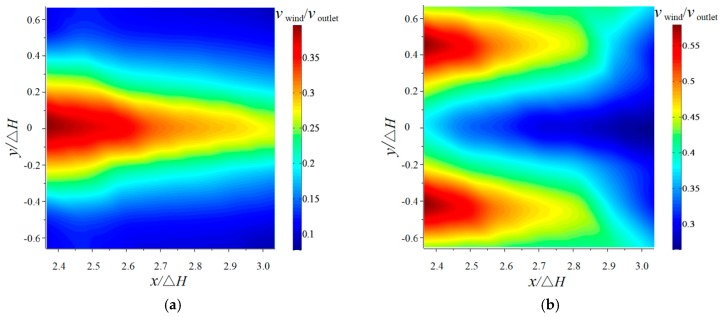
The nappe wind distribution of the two-dimensional plane (*z*/Δ*H* =0): (**a**) CB and (**b**) TB.

Since the flip bucket is axisymmetric, the velocity of the downstream nappe wind is measured on a side of the bucket axis. The horizontal distributions (*z*/Δ*H* = 0) of the downstream nappe wind for the CB and TB with an angle of 50° are shown in Figure 7. The experimental results show that the velocities of the wind for the CB and TB both decrease with the increase in *x* under different *y* values, but the maximum velocity of the wind for the TB is bigger than that of the CB. For the CB, the nappe wind rapidly decreased with the movement distance of the nappe, and the peak value is on the bucket axis. The influence width of the nappe is ±0.448 from the axis of the bucket. The lateral distribution of the nappe wind obeys a normal distribution due to the constant impingement angle of the trajectory nappe, which is consistent with the research by Liu [10]. For the TB, the velocity of the wind also decreases with the movement distance of the nappe. The shape of the lateral distribution is similar to a hump, and the peak value is when *y*/Δ*H* is ±0.448 on the outside of the bucket axis. The influence width of the nappe is ±0.672 bigger than that of the CB. The nappe wind is generated by the additional momentum from the high-speed nappe with the dragging force between the breakup nappe or the splash and the surrounding air. Compared with the CB’s nappe, the TB’s nappe appears to have a strong breakup. The greater breakup of the nappe could cause the increase in splashes, and then the momentum exchange between the water and air is more intensive [23,27]. It causes the velocity of the wind for the TB to obviously enhance with the increase of the *y* value.

#### 3.2.2. Vertical Distribution

**Figure 8:**
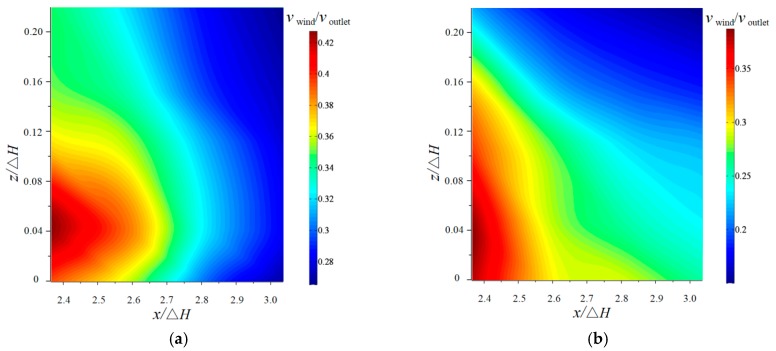
Vertical distribution of velocity of the nappe wind (*y*/Δ*H* =0): (**a**) CB, and (**b**) TB.

The vertical distribution of the nappe wind for the CB and TB under different *x* values when *y*/Δ*H* is 0 is shown in Figure 8. The experimental results clarify that both wind velocities rapidly decrease with the increase in the *x* direction under different heights. For the CB, the nappe wind first tends to increase slightly, and then decrease as *z* increases. The main reason is that the effect of the trajectory nappe and the splashing droplets is reduced with the increase of the vertical height. The peak value of the nappe wind velocity appears when *z*/Δ*H* is 0.045 due to the friction between the water surface and the nappe wind [23]. When *y*/Δ*H* is 0, the nappe wind of the CB is slightly larger than that of the TB due to the surface boundary effect and the vortex in the air [27]. For the TB, when *x*/Δ*H* is 2.37, the trend of wind velocity is similar to that of the CB. However, the other trends are such that the velocity deceases as *z* increases, which is the result of the turbulent friction between the breakup nappe and the sounding air.

**Figure 9:**
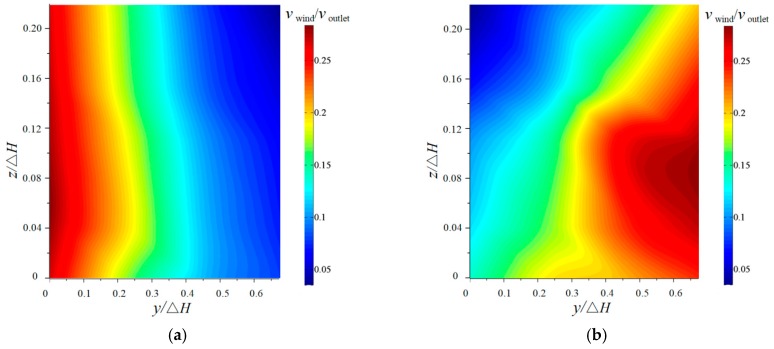
Vertical distribution of velocity of the nappe wind (*x*/Δ*H*=3.04): (**a**) CB and (**b**) TB.

Figure 9 depicts the vertical distribution of the downstream nappe wind under different *y* values when *x*/Δ*H* is 3.04 as an example in order to analyze the effect of bucket shapes on the wind velocity. For the CB, the nappe wind velocity decreases slightly when *y*/Δ*H* is 0.448 and 0.672, but it increases slightly first and then decreases along the vertical direction when *y*/Δ*H* is 0 and 0.224. The friction of the surface boundary is more significant when the nappe wind velocity is large, which results in the nappe wind near the ground being relatively small [23]. For the TB, the nappe wind decreases along the vertical direction when *y*/Δ*H* is 0 and 0.224. However, the nappe wind velocity increases first and then decreases when *y*/Δ*H* is 0.448 and 0.672, which is the result of the breakup nappe. When *y*/Δ*H* is between 0–0.672, the nappe has fewer breakups than when *y*/Δ*H* is between 0.448–0.672. The air entrainment and turbulence of the nappe could enhance the momentum exchanged between the air and water, and cause the greater wind velocity.

### 3.3. Effects of Bucket Angles on Wind Velocity

#### 3.3.1. Horizontal Distribution

**Figure 10:**
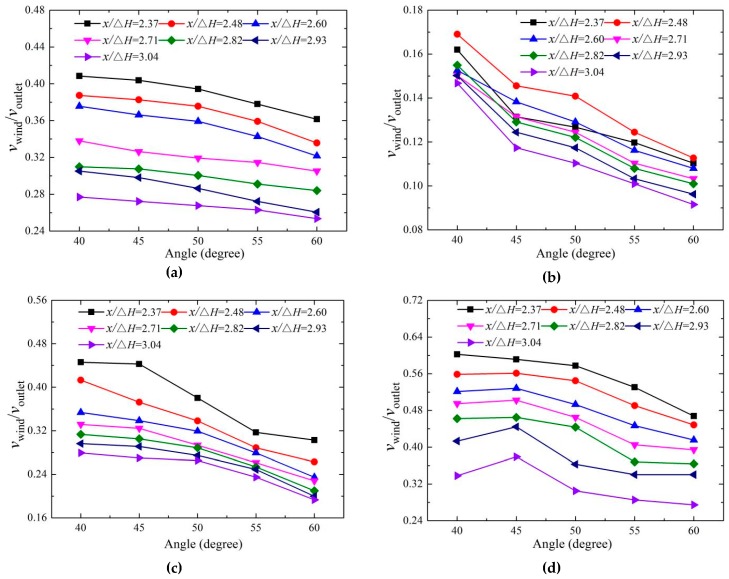
The two-dimensional distribution of the nappe wind for different bucket angles at *z*/Δ*H* = 0: (**a**) CB (*y*/Δ*H* = 0), (**b**) CB (*y*/Δ*H* = 0.448), (**c**) TB (*y*/Δ*H* = 0), and (**d**) TB (*y*/Δ*H* = 0.448).

The bucket angle has a great influence on the flow regime of the trajectory nappe, and then it strongly affects the velocity distribution of the downstream nappe wind. The horizontal distribution of the nappe wind is measured under five different bucket angles when *z*/Δ*H* is 0. The velocities when *y*/Δ*H* is 0 and 0.448 respectively appear as the peaks for two buckets, so the distributions at two points are shown in Figure 10.

The test results indicate that for the CB and TB, the velocity of the wind decreases with the increase of the bucket angle and the *x* value when *y* is 0, and the results are similar to the length of the nappe. When *y* is 0, the test point is located at the downstream of the nappe core, and the nappe core has less breakup and air entrainment [20]. When *y*/Δ*H* is 0.448, the wind velocity of both buckets decreases as the angle increases near the impinging region, but the wind velocity of the TB first increases and then decreases as the bucket angle increases when the test point is far away from impinging area, and the velocity peaks at 45° angles. The main reason is that when the bucket angle is 40° and *x*/Δ*H* is between 2.93–3.04, the impingement width is smaller than that of the other bucket angles, which causes the trajectory nappe to have a relatively narrow range of action and less momentum.

#### 3.3.2. Vertical Distribution

**Figure 11:**
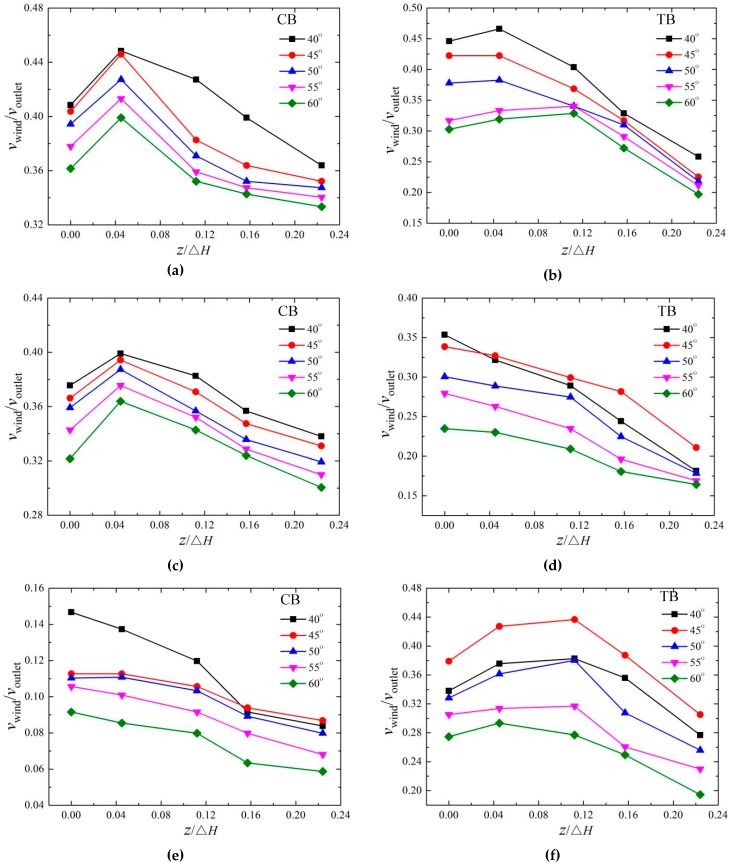
The wind velocity of the vertical distribution at different bucket angles: (**a**) CB (2.37, 0), (**b**) TB (2.37, 0), (**c**) CB (2.60, 0), (**d**) TB (2.60, 0), (**e**) CB (3.04, 0.448), and (**f**) TB (3.04, 0.448).

The vertical distribution of the wind velocity for the CB and TB with different bucket angles is measured. Figure 11 depicts the vertical distribution of the wind velocity for the CB and TB with different angles at three test points of (2.37, 0), (2.60, 0), and (3.04, 0.448). For the CB, the velocity of wind decreases with the increasing angles. When *y* is 0, the vertical distributions of the wind velocity of the CB at the five angles increase slightly, and then decrease with the increasing height; the peak exists when *z*/Δ*H* is 0.045. The velocity when *z* is 0 is smaller than that when *z*/Δ*H* is 0.045 due to the friction between the water surface and the surrounding air [23,27]. The trajectory length has a great influence on the distribution of the nappe wind on the bucket axis (*y*/Δ*H* = 0). The nappe wind is caused by the dragging force of the nappe and the splashing in the air [27].

For the TB, the velocity of the wind decreases with the increase of the bucket angles when (*x*/Δ*H, y/*Δ*H*) is (2.37, 0) and the velocity peak moves downstream as the angles increase. However, when the test point moves to (2.60, 0), the velocity at 45° starts to be bigger than that at 40°. When the test point is at (3.04, 0.448), the velocity at 45° is biggest, and a peak for all the angles exists when *z*/Δ*H* is 0.112; the results are caused by the shape of the trajectory nappe and the impinging area [14,29].

The trajectory length decreases with the increase of the bucket angles, the trajectory width, and the height. The smaller the bucket angle is between 40°–60°, the larger the trajectory length, and the stronger the nappe wind. However, when *y*/Δ*H* is larger than 0, the distribution of the nappe wind on both sides of the bucket axis is mainly determined by the impinging edge width. When the bucket angle is 40°, the impinging edge width is relatively small, which makes the nappe wind smaller than that when the angle is 45°. When the viscous force and frictional force decrease, the nappe wind is attenuated accordingly.

### 3.4. Splash Characteristics

#### 3.4.1. Effects of Bucket Shape

**Figure 12:**
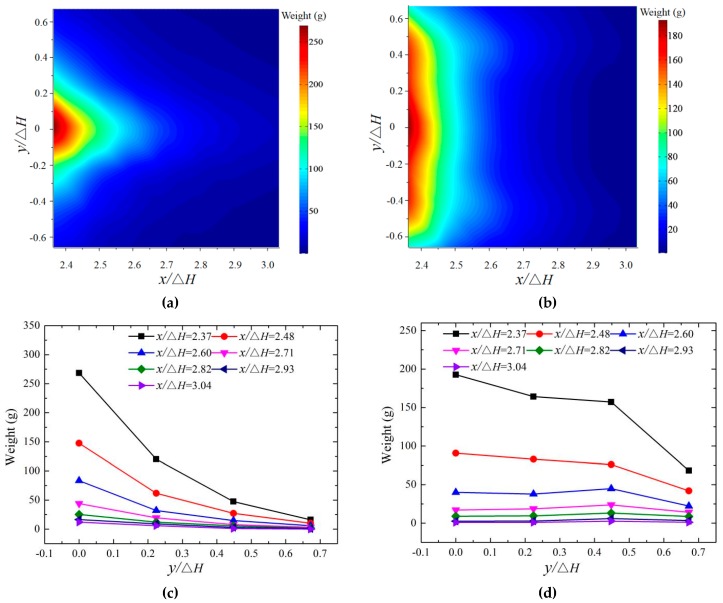
The downstream distribution of the splashing droplets (z/Δ*H* = 0): (**a**,**c**) CB, (**b**,**d**) TB.

The downstream nappe wind mainly consists of the dragging wind, diffusing wind, and splashing wind, and the splashing droplets have an important influence on the wind velocity in this study. The intensive interaction between the rapid splashing droplets and air would cause the strong nappe wind within a certain range, and the nappe wind would cause the splashing droplets to spread further. This is because the powerhouse, booster station, and owner camp are mostly arranged on the ground in practical engineering, and the splash distribution on the ground is very crucial to the operation safety of the dam. The splashing weight is measured using sponge collection boxes. The boxes are weighed using an electronic scale, and they are collected 80 min later; then, the average weight of the splashing droplets is obtained by repeating the measurements. The weights of the downstream splash for the CB and TB are shown in Figure 12. The test results prove that for the CB and TB, the splashing weight rapidly decreases as *x* increases. For the CB, the lateral distribution of the splash perpendicular to the flow direction obeys a normal distribution that is similar to that of the nappe wind. The axial distribution of the splashing droplets along the flow direction conforms to a gamma distribution, which is consistent with the previous research [7,31]. The peak value of the droplets appears on the axis of the bucket, and the splashing droplets rapidly decrease as the distance between the measuring point and the bucket axis increases. The main reason for this is that the trajectory nappe of the CB is more concentrated, and the impingement width is relatively small. In addition, the maximum weight is on the bucket axis, which results in the velocity of the wind on the bucket axis being much larger than that on either side.

The splashing weight of the CB is much larger than that of the TB on the bucket axis, but the weight for the TB is larger than that of the CB on both sides. As *y* increases, the weight of the droplets for the CB strongly decrease, but for the TB, the decease trend is slighter when the test point is close to the impinging area. When the test point is far from the impinging area, the weight is steady. The main reason for this is that the trajectory nappe of the CB is more concentrated than that of the TB, and the unit width discharge and the splashing droplets are both larger than those of the TB [16,29]. For the TB, the splashing droplet slightly decreases as *y* increases near the impingement position (*x*/Δ*H* is 2.37 and 2.48). Many large-sized splashing droplets near the impingement position are hardly affected by the nappe wind. As the droplet diameter increases, the ejection requires a larger initial impulse, and the splash velocity and splash area are relatively small. The weight of the splashing droplets on the bucket axis is much larger than that on both sides. When *x*/Δ*H* is larger than 2.60 (far away from the impingement position), the weight is steady, and the splashing droplets mainly consist of small diameter droplets that are greatly affected by the nappe wind. The distribution of the splashing droplet is similar to the nappe wind distribution. The weight of the splash droplets at different vertical heights is a valuable subject that will be further studied in the future.

#### 3.4.2. Effects of Bucket Angles

**Figure 13:**
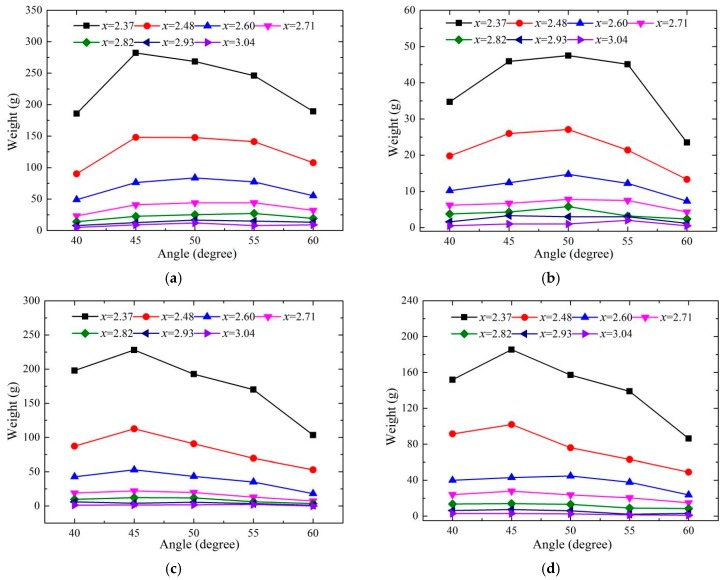
The distribution of the droplet weights for different bucket angles: (**a**) CB (*y*/Δ*H* = 0), (**b**) CB (*y*/Δ*H* = 0.448), (**c**) TB (*y*/Δ*H* = 0), and (**d**) TB (*y*/Δ*H* = 0.448).

The distributions of the splashing water droplets with the different bucket angles when *y* is 0 and 0.448 are shown in Figure 13. The distributions of the splashing droplets for the CB and the TB are similar at the test area, and the weight of the splashing droplets first increases and then decreases with the increase in the bucket angles. The peak value appears at the angle of 45° when the weight of the droplets is larger than 100 g. When the weight is smaller than 100 g, the relationship between the weight and the angles is not obvious. The trajectory length decreases slightly, but its impingement width increases significantly when the angle changes from 40° to 50°. The weight of the splashing droplets on both sides is affected by the comprehensive influence of the trajectory length and impinging width. The weight of the splashing droplets on the bucket axis is up to the trajectory height and the trajectory length of the nappe.

## 4. Conclusions

The downstream nappe wind intensively affects the rainfall intensity and distribution, the ecological environment, and the operational safety of the dam. A physical experiment was conducted to study the spatial distribution of the downstream nappe wind and the splash for the CB and TB with five bucket angles. The research results indicate the following.

(1) The nappe of the CB has a concentration similar to a crescent, and the width of the nappe is the same as the width of the bucket. The nappe of the TB is diffused similar to a fan, and the width of the nappe is bigger than that of the CB due to the smaller length of the sidewalls and the circular edge of the TB. The maximum trajectory length decreases as the angles of the buckets increase. Conversely, the maximum trajectory height and width increase.

(2) The horizontal distribution of the downstream nappe wind in the TB is quite different from that of the CB. For the CB, the velocity of the wind rapidly decreases with the increasing *x* or *y* and obeys a normal distribution in the lateral distribution. Meanwhile, for the TB, the velocity of the wind also decreases with the increasing *x*. With the increasing *y*, the peak value exists when *y*/Δ*H* is 0.448, and is similar to a hump in the lateral distribution. The nappe wind of the CB is slightly larger than that of the TB on the axis, while that of the CB is generally smaller than that of the TB on the outsides. The velocities of the nappe winds result from the shapes of both buckets, and the diffusion of the nappe for the TB enhances its air entrainment and turbulence.

(3) Along the axis of buckets, the velocity of the nappe wind for the CB increases and then reduces as *z* increases, and the velocity peaks when *z*/Δ*H* is 0.045. For the TB, the velocity decreases as *z* increases along the axis of the bucket, and the velocity on the outsides of the bucket axis peaks when *z*/Δ*H* is 0.112 and *x*/Δ*H* is 3.04. The velocity of the wind results from the trajectory length, width, and height.

(4) The velocity of the wind decreases as the angles for the CB increase. For the TB, a peak appears at 45° as the angles increase. The velocity of the wind is caused by dragging force between the surrounding air and the nappe or the splash. The results are on the basis of the shape of the nappe and splash.

(5) The intension of the splashing water droplets and the velocity of the nappe wind have mutual effects. For the CB and TB, the splashing weights both decrease strongly with the increasing *x*, and increase and then decrease with the increasing angles, and the maximum appears at 45°. However, the splash weight of the CB is much larger than that of the TB near the axis of the bucket, and the opposite result is obtained on the outsides, which is the one of reasons for the velocity distribution. For the TB, the splashing weight decreases as the *y* value increases near the impinging position (*x*/Δ*H* is from 2.37 to 2.48), while the weight is stable when the test point is far away from the impinging position (*x*/Δ*H* is from 2.60 to 3.04).

This research result can accurately predict rainfall distribution and ensure the operational safety of the dam and improve the surrounding ecological environment. The vector field of the nappe wind and the similarity law will be further studied in future research.
